# A Comparative Analysis of Risk Perception and Coping Behaviors among Chinese Poultry Farmers Regarding Human and Poultry Infection with Avian Influenza

**DOI:** 10.3390/ijerph16203832

**Published:** 2019-10-11

**Authors:** Bin Cui, Feifei Wang, Linda Dong-Ling Wang, Chengyun Pan, Jun Ke, Yi Tian

**Affiliations:** 1 Business College of Yangzhou University, Yangzhou 225001, Jiangsu Province, China; yzdxwff@126.com (F.W.); pcypcy22@163.com (C.P.); jke@yzu.edu.cn (J.K.); Jstianyi@163.com (Y.T.); 2 Key Laboratory of Prevention and Control of Biological Hazard Factors (Animal Origin) for Agrifood Safety and Quality, Ministry of Agriculture of China, Yangzhou University, Yangzhou 225001, Jiangsu Province, China; 3 Institute of Translational Medicine, Medical College, Yangzhou University, Yangzhou 225001, Jiangsu Province, China; lindawdl@yzu.edu.cn; 4 Jiangsu Key Laboratory of Experimental & Translational Non-Coding RNA Research, Yangzhou 225001, Jiangsu Province, China

**Keywords:** Avian influenza, coping behavior, Chinese poultry farmer, comparative analysis, risk perception

## Abstract

Poultry farmers face a dual risk when mutant avian influenza (AI) virus exhibits zoonotic characteristics. A/H5N1 and A/H7N9 are two principal strains of the AI virus that have captured public attention in recent years, as they have both been reported and can infect poultry and humans, respectively. Previous studies have focused either on poultry farmers’ risk perception and biosecurity preventive behaviors (BPBs) against A/H5N1 infection with poultry, or on their risk perception and personal protective behaviors (PPBs) against human infection with A/H7N9, even though these two strains often appear simultaneously. To bridge this research gap, a survey (*N* = 426) was conducted in the Chinese provinces of Jiangsu and Anhui to assess risk perception and coping behaviors adopted by poultry farmers facing the dual risk of these two viral strains. Paired sample *t*-tests revealed that farmers’ perceived risk of poultry infection with A/H5N1 was significantly higher than their perceived risk of human infection with A/H7N9, and that their reported frequency of BPBs against A/H5N1 was significantly higher than the frequency of PPBs against A/H7N9. Moreover, farmers were less familiar with AI infection in human beings compared to that in poultry, but they felt a higher sense of control regarding human AI infection. Multivariate regression analyses showed that farm size and perceived risks of both human and poultry infection with AI were positively associated with BPBs and PPBs. The findings of this research suggest that a campaign to spread knowledge about human AI infection among poultry farmers is needed, and that a policy incentive to encourage large-scale poultry farming could be effective in improving implementation of BPBs and PPBs.

## 1. Introduction

Avian influenza (AI) has captured the attention of the public in recent years, as it is highly contagious in poultry and can infect humans [1]. Reports have shown that the AI H5N1 virus (A/H5N1) has led to the culling of more than 40 million poultry since 2004 and has also consequently led to large economic losses in the poultry industry in mainland China [2]. In addition, a total of 1567 laboratory-confirmed cases of humans infected with AI H7N9 virus (A/H7N9), including at least 615 deaths, were reported in China from early 2013 to 5 September 2018 [3].

The China Animal Industry Yearbook (2017) reported that in 2016, more than 29 million chicken farmers had flocks of less than two thousand birds, compared to fewer than 500,000 with flocks in excess of two thousand birds [4]. Owing to daily contact with poultry and in light of the zoonotic characteristics of the AI virus, poultry farmers face a dual risk. Specifically, poultry infected with A/H5N1 may bring about economic losses, and poultry farmers themselves may also contract A/H5N1 and A/H7N9 [5,6] due to their long-term exposure to poultry [7].

A large body of research has examined the problems that poultry farmers face regarding AI. This work has tended to focus either on farmers’ risk perception and biosecurity preventive behaviors (BPBs) against A/H5N1 infection in poultry [8,9,10] or on the risk perception and personal protective behaviors (PPBs) of poultry workers [11], live poultry traders [12], and poultry farmers [13] against human infection with A/H7N9. To the best of our knowledge, no published studies have examined both of these factors together, even though human infection is often accompanied by poultry infection and the resulting risk of economic loss.

The current study measured risk perception and coping behaviors adopted by Chinese poultry farmers responding to AI infection and facing the dual risk of economic losses and human infection.

## 2. Methods

### 2.1. Study Location and Sampling

The Yangtze River Delta, located in Eastern China, includes the provinces of Shanghai, Jiangsu, Anhui, and Zhejiang, and is among the areas at highest risk for AI epidemics in China [14,15]. Jiangsu and Anhui provinces were chosen as sites for the present study, as they account for the highest proportion of egg and poultry production in the Yangtze River Delta, at 49.7% (3.18 million tons) and 40.7% (2.61 million tons), respectively [4]. In addition, the geographic proximity of these provinces to the research team as well as researchers’ familiarity with local dialects maximized ease of access and communication. Study participants were commercial chicken farmers whose main source of income was chicken farming. Unlike chicken farm employees and subsistence farmers who raise a small number of chickens in their backyard, commercial chicken farmers must make all management decisions and take full responsibility for their profits and losses. Chicken production is the most common form of poultry farming in China [4], and AI viruses are known to cause severe infection in chickens [16]. Accordingly, in the interest of consistency and to avoid the influence of the risk perceptions of farmers of other poultry species, only chicken farmers were included in this study.

**Figure 1:**
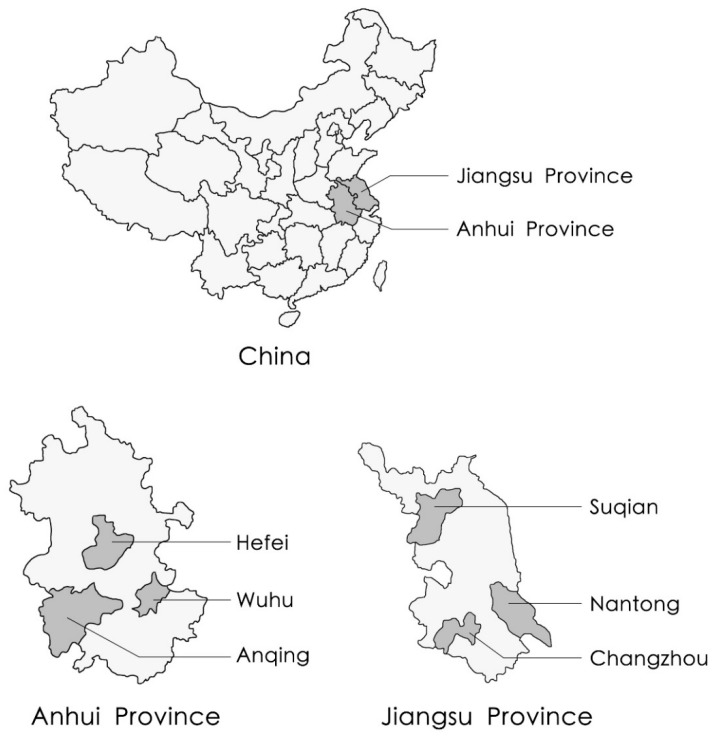
Map of the study areas in China.

Potential study participants were identified using mixed stratified sampling and random sampling. First, at the prefectural level, three cities in each province were randomly selected (Changzhou, Suqian, and Nantong in Jiangsu province, and Hefei, Wuhu, and Anqing in Anhui province, see Figure 1). Second, two county-level units were randomly selected within each of these cities. Third, two districts were randomly selected within each county-level unit. Fourth, two villages were randomly selected from each selected district. Finally, 7–10 chicken farmers within each selected village were randomly selected from the lists of names provided by local veterinary bureaus. Six research assistants underwent training designed to minimize bias and then conducted face-to-face interviews with the selected chicken farmers using a standardized questionnaire. Of 450 farmers invited to participate in the study, a total of 426 completed interviews, resulting in a response rate of 94.7%. Only 24 farmers declined to participate, citing lack of time or other unstated reasons.

### 2.2. Ethics, Consent, and Permission

The survey was conducted from February to May 2017 following ethical approval from Yangzhou University and the local official veterinary bureaus of the six cities included in the study. Official veterinary bureaus have the primary responsibility for distributing AI prevention guidelines and monitoring AI in mainland China. Potential survey participants were first provided with an explanation of the research and asked for their consent to participate. Those who agreed to participate completed a face-to-face interview using a standardized questionnaire. The questionnaire was fully anonymous, and no personal identifying information was collected.

### 2.3. Study Instrument

The questionnaire was divided into three sections (see Appendix A). The first part contained questions about socio–economic information, including gender, age, educational level, number of years of experience raising chickens, farm operation size (measured by amount of livestock on hand at the time of the survey), and whether or not the respondent’s poultry had ever previously been infected with AI.

The second part of the survey contained questions to measure farmers’ risk perception regarding human infection with A/H7N9 and poultry infection with A/H5N1. We measured risk perception characteristics using a framework of familiarity and controllability, based on Slovic’s study of risk perception [17]. Specific risk events examined included route of transmission and infectivity, reason for occurrence, prevention measures taken, cure rate, death rate, and overall feelings about human infection with A/H7N9 and poultry infection with A/H5N1. For each risk perception characteristic, respondents were asked to indicate their level of agreement or disagreement with corresponding statements for familiarity and controllability on a 5-point Likert-type scale (ranging from “1 = totally unfamiliar” or “totally out of control” to “5 = very familiar” or “completely under control”). Farmers’ perceived risk regarding human infection with A/H7N9 and poultry infection with A/H5N1 was measured on a 5-point Likert-type scale ranging from “1 = no perceived risk whatsoever” to “5 = very high perceived risk”.

The third part of the survey measured actual coping behaviors and included nine questions about biosecurity preventive behaviors (BPBs) considered to be the first line of defense against poultry infection with A/H5A1 [18], as well as other BPBs that have been shown to be effective in preventing and controlling the spread of A/H5A1 at the farm level [19]. The survey also included seven questions about human infection with A/H7N9 recommended by the National Health and Family Planning Commission of China in its proposal for personal protective behaviors (PPBs) against AI for high-risk persons including poultry farmers, aiming to reduce their risk of contracting AI viral infection due to occupational exposure to poultry [13]. Respondents were asked to report the frequency with which they had adopted each coping behavior in their routine husbandry practices. Responses were on a 5-point Likert-type scale ranging from “1 = never adopted” to “5 = adopted all the time”.

### 2.4. Data Analysis

The paired sample *t*-test was used to compare poultry farmers’ risk perception characteristics regarding human infection with A/H7N9 with those for poultry infection with A/H5N1, and to compare farmers’ PPBs against A/H7N9 (sum all of reported PPB frequencies) with BPBs against A/H5N1 (sum all of reported BPB frequencies). A descriptive statistical method was applied to present the percentage of farmers reporting each coping behavior against human infection with A/H7N9 and poultry infection with A/H5N1.

Linear regression analysis was used to examine the determinants associated with adoption of BPBs and PPBs. The nine items for BPBs and seven items for PPBs were each standardized by summing the scores for the items making up each factor, subtracting the mean value, and dividing by the standard deviation. The standardized BPB and PPB scores were then used as dependent variables in the regression analyses, with socio–economic variables and risk perception characteristics used as independent variables. The criterion for statistical significance was *P* ≤ 0.05.

## 3. Results

Demographic characteristics of the 426 chicken farmers included in the study are presented in Table 1. Of the respondents, 25.1% had previous experience of chickens being infected with A/H5N1. Medium-scale farms were the most common in the study, with 56.6% of farms rearing between 1001 and 10,000 chickens and 21.8% with more than 10,000.

**Figure 2:**
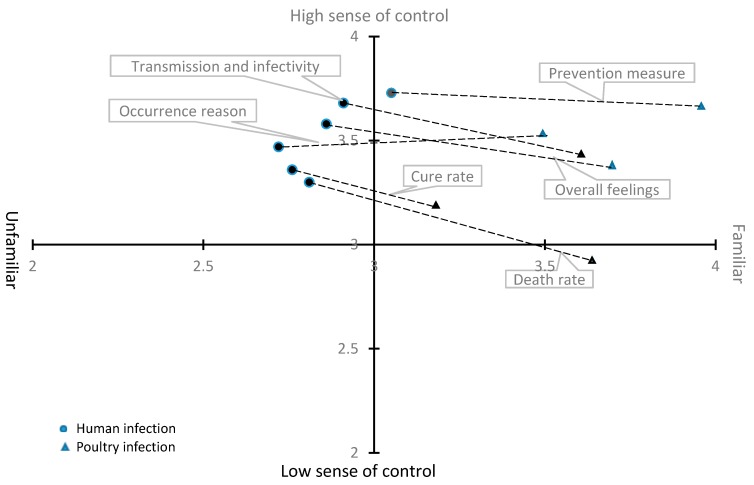
Spatial map of risk perception characteristics regarding human infection and poultry infection.

Risk perception characteristics for human and poultry infection are shown in Figure 2. Perceived risks of transmission and infectivity, occurrence reason, cure rate, death rate, and overall feelings about human infection with A/H7N9 were in the quadrant of “high sense of control” and “unfamiliar”. Perceived risks of transmission and infectivity, occurrence reason, cure rate, and overall feelings about poultry being infected with A/N5N1 were in the quadrant of “high sense of control” and “familiar”. Only death rate from poultry infection was in the quadrant of “low sense of control” and “familiar”. None of the characteristics were in the quadrant of “low sense of control” and “unfamiliar”.

Respondents’ perceived risk of poultry infection was significantly higher than their perceived risk of human infection (T = 12.97, *P* < 0.001; Table 2). Respondents’ familiarity with occurrence reason, transmission and infectivity, cure rate, prevention measure, death rate, and overall feelings about poultry infection were significantly higher than their familiarity with human infection. However, respondents’ sense of control about transmission and infectivity, death rate, and overall feelings about poultry infection were significantly lower than their sense of control about human infection. The total frequency of BPBs against A/H5N1 was significantly higher than the frequency of PPBs against A/H7N9 (T = 23.89, *P* < 0.001).

**Figure 3:**
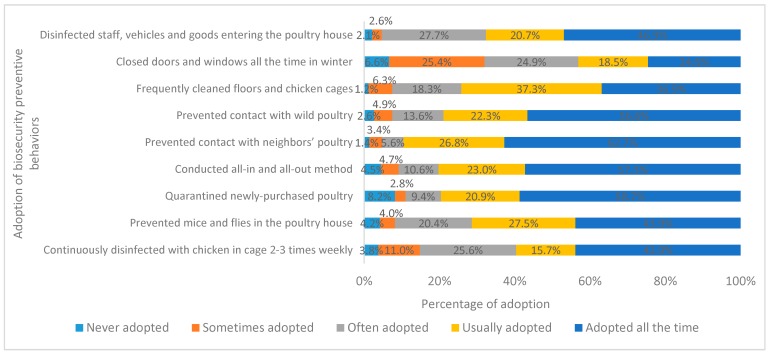
Percentage of respondents reporting adoption of biosecurity preventive behaviors against avian influenza (AI).

**Figure 4:**
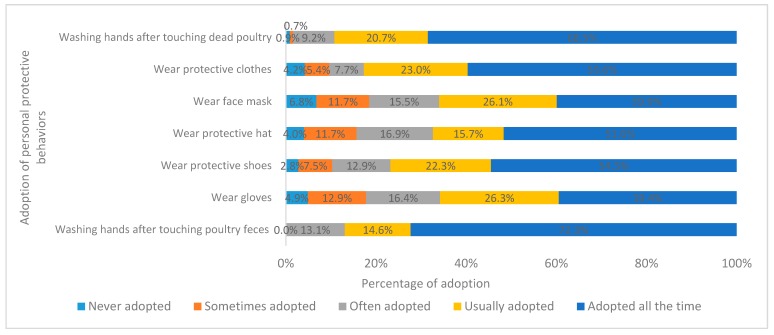
Percentage of respondents reporting adoption of personal protective behaviors against AI.

The rates of reported adoption of BPBs and PPBs are shown in Figure 3 and Figure 4, respectively. The proportion of respondents who reported always adopting “prevented contact with neighbors’ poultry” (62.7%), “quarantined newly-purchased poultry” (58.7%), “conducted all-in and all-out method” (57.3%), and “prevented contact with wild poultry” (56.6%) was higher than that of the other BPBs. Of note, 8.2% of poultry farmers had never adopted “quarantined newly-purchased poultry”, and 6.6% had never adopted “closed doors and windows all the time in winter” (Figure 3).

The proportion of respondents who had adopted “washing hands after touching poultry feces” (72.3%), “washing hands after touching dead poultry” (68.5%), and “wearing protective clothes” (59.6%) was higher than that of the other PPBs. Of note, 6.8% of poultry farmers had never adopted “wearing face mask” and 4.9% had never adopted “wearing gloves” while working (Figure 4).

Results of the multivariate linear regression are shown in Table 3. Male gender and age were significantly and negatively associated with PPBs. More years of experience raising chickens and larger farming operation size were significantly and positively associated with both BPBs and PPBs, while experience with infected poultry was significantly and negatively associated with both BPBs and PPBs. Risk perception of poultry infection and human infection were significantly and positively associated with BPBs and PPBs, respectively.

## 4. Discussion

This study examined Chinese poultry farmers’ risk perception and coping behaviors regarding human infection with A/H7N9 and poultry infection with A/H5N1. The study found that farmers’ perceived risk of poultry being infected with A/H5N1 was significantly higher than their perceived risk of human infection with A/H7N9, and that their total frequency of BPBs against A/H5N1 was significantly higher than the frequency of PPBs against A/H7N9. These findings reflect a positive correlation between risk perception and coping behavior regarding AI. Moreover, farmers were less familiar with human A/H7N9 infection than with A/H5N1 infection in poultry.

A/H5N1 and A/H7N9 are among the most important AI viruses responsible for recent outbreaks in China. A/H5N1 is pathogenic in poultry, while A/H7N9 is not; both viruses have caused disease in humans, but the number of human A/H7N9 cases far exceeds that for A/H5N1 [20]. The A/H5N1 virus has infected poultry in mainland China since 2004 [2]. However, only after China changed its infectious disease policy in 2013 [21] was information on human infection with A/H7N9 made routinely available to the public [22,23]. Therefore, A/H7N9 was a previously unknown virus for Chinese poultry farmers, but the long-term practice of combatting the infection of poultry with A/H5N1 and the substantial economic losses previously suffered have made Chinese poultry farmers more familiar with that virus. However, genomic analysis suggests that the A/H7N9 virus is of avian origin and is mainly transmitted through exposure to infected poultry [24]; thus, poultry farmers are at an elevated risk of contracting the AI virus [5]. It has been reported that some poultry workers have been infected with A/H7N9 [7]. Therefore, it is important that knowledge of human AI infection be disseminated among poultry farmers.

Survey respondents’ sense of control regarding transmission and infectivity, death rate, and overall feelings about human infection were significantly higher than their sense of control regarding poultry infection. Existing research shows that live poultry purchasing practices currently employed in China increase the risk of exposure to A/H7N9 virus-contaminated environments [25,26]. It was reported that live poultry markets (LPMs) were the predominant source of influenza A/H7N9 exposure in China, and that the closing of LPMs reduced the incidence of human A/H7N9 infection by 97–99% [27]. Closure of LPMs most likely stopped A/H7N9 transmission from these locations, which would also increase poultry farmers’ sense of control regarding human infection.

Consistent with our previous surveys on adoption of BPBs [10] and PPBs [13] among Chinese poultry farmers, the present study further clarified the frequency of adopting different BPBs and PPBs from “never adopted” to “adopted all the time”. Consistent with previous research among Chinese poultry and broiler farmers [8,10,28], those who manage larger farms and those with more years of experience raising poultry were likely to report greater adoption of BPBs and PPBs. The present study found that farmers who had experience with poultry infection were less likely to report adopting BPBs and PPBs. This pattern may be related to the length of time since the poultry infection, as over time farmers may have forgotten the experience of infection and the effect it had on their livelihood and livestock. However, the factors underlying these patterns require further study.

This study found that poultry farmers who had higher risk perception regarding human AI infection reported higher rates of PPB adoption. This finding is consistent with Protection Motivation Theory (PMT) [29] and with the Health Belief Model (HBM) [30], both of which have been applied to understanding personal protective health behaviors. However, the study further found that poultry farmers with higher risk perception regarding poultry infection with AI also reported higher rates of BPB adoption. These findings suggest that the principles of PMT and the HBM could be extended to the field of economic threat analysis and could be applied to understand and predict personal economic behaviors (i.e., avoiding economic loss). Further study is warranted to determine whether the mechanism of influence for BPBs is similar to that for personal protective health behaviors.

Risk perception and coping behaviors were measured using respondent self-reports. This may have led to a discrepancy between actual and reported risk perception and behaviors. However, the study survey was conducted anonymously, and we did not ask personal or sensitive questions. Therefore, we do not expect that our study would be subject to a high degree of information bias.

## 5. Conclusions

In this study comparing Chinese poultry farmers’ risk perception regarding AI virus infection in poultry and humans with their coping behaviors, we found that farmers’ perceived risk of poultry infection with A/H5N1 was significantly higher than that for human infection with A/H7N9, and that the total frequency of reported BPBs against A/H5N1 was significantly higher than the frequency of PPBs against A/H7N9. Moreover, farmers were less familiar with human infection with A/H7N9 compared to poultry infection with A/H5N1, likely because they were unfamiliar with the A/H7N9 virus. Nevertheless, farmers felt a greater sense of control about human AI infection, presumably because the closure of LPMs greatly reduced or eliminated AI transmission in China. Accordingly, knowledge translation (for example, specially organized training, distribution of brochures) regarding human AI infection is needed in this population.

The size of poultry farming operations was associated with BPBs and PPBs. A larger farm size usually implies greater overall economic losses if poultry are infected, forcing larger-scale farmers to improve their poultry management standards [10] including personal protective management standards. This suggests that larger farm size is conducive to poultry farmers’ adoption of actual BPBs and PPBs, whereas a smaller farm size may increase the risk of human infection with A/H7N9 and poultry infection with A/H5N1 in China. Thus, a policy incentive to encourage large-scale poultry farming could be effective in improving the implementation of both BPBs and PPBs, thereby reducing infection rates.

Finally, poultry farmers’ risk perception regarding both human and poultry infection with AI was associated with BPBs and PPBs, suggesting that risk perception can lead to the adoption of preventive measures. Previous studies have shown that perceived risk is often triggered by the availability and quality of risk information [31]. Therefore, dissemination of accurate information about AI risks is crucial. Further study is needed to determine the most effective knowledge translation strategies.

## Figures and Tables

**Table 1:** Respondent characteristics (*N* = 426).

Characteristics	*N*	%
Gender		
Female	99	23.2
Male	327	76.8
Age		
≤35 years	11	2.6
36–45 years	112	26.3
46–55 years	180	42.3
≥56 years	123	28.9
Education		
Primary or below	129	30.3
Junior high school	213	50.0
Senior high school	77	18.1
Three-year college	5	1.2
Undergraduate college or above	2	0.5
Years raising poultry		
≤5 years	128	30.0
6–10 years	151	35.4
11–15 years	69	16.2
16–20 years	37	8.7
21–25 years	21	4.9
26–30 years	18	4.2
≥31 years	2	0.5
Experience with previous infection		
Previous A/H5N1 chicken infection	107	25.1
No previous A/H5N1 chicken infection	319	74.9
Farming scale		
≤300 chickens	38	8.9
301–1000 chickens	54	12.7
1001–10,000 chickens	241	56.6
≥10,001 chickens	93	21.8

**Table 2:** Comparisons of risk perception characteristics regarding human infection with A/H7N9 and poultry infection with A/H5N1.

Paired Sample (Poultry Infection—Human Infection)	Paired Differences		*P* Value
	Mean	Standard Deviation	
Risk perception of poultry infection—Risk perception of human infection	0.514	0.818	0.000
Familiarity of occurrence reason to poultry infection—Familiarity of occurrence reason to human infection	0.754	1.308	0.000
Familiarity of route of transmission and infectivity to poultry infection—Familiarity of route of transmission and infectivity to human infection	0.676	1.249	0.000
Familiarity of cure rate to poultry infection—Familiarity of cure rate to human infection	0.415	1.450	0.000
Familiarity of prevention measure to poultry infection—Familiarity of prevention measure to human infection	0.859	1.429	0.000
Familiarity of death rate to poultry infection—Familiarity of death rate to human infection	0.793	1.397	0.000
Familiarity of overall feelings about poultry infection—Familiarity of overall feelings about human infection	0.800	1.152	0.000
Controllability of poultry infection—Controllability of human infection occurrence	0.082	1.367	0.215
Controllability of route of transmission and infectivity to poultry infection—Controllability of route of transmission and infectivity to human infection	−0.223	1.358	0.001
Controllability of cure rate to poultry infection—Controllability of cure rate to human infection	−0.136	1.446	0.053
Controllability of prevention measure to poultry infection—Controllability of prevention measure to human infection	−0.054	1.294	0.390
Controllability of death rate to poultry infection—Controllability of death rate to human infection	−0.322	1.497	0.000
Controllability of overall feelings about poultry infection—Controllability of overall feelings about human infection	−0.178	1.286	0.004

*P* values are from paired sample *t*-tests; statistical significance was set at *P* ≤ 0.05.

**Table 3:** Multiple regression coefficients (standardized β, standard error) for socio–economic and risk perception variables associated with adoption of biosecurity preventive behaviors and personal protective behaviors.

Predictor Variables	Biosecurity Preventive Behaviors					Personal Protective Behaviors				
	β	SE	95% Confidence Interval		VIF	β	SE	95% Confidence Interval		VIF
			Lower Bound	Upper Bound				Lower Bound	Upper Bound	
Gender (Male)	0.001	0.101	−0.197	0.201	1.103	−0.122**	0.111	−0.507	−0.072	1.103
Age	0.011	0.058	−0.101	0.129	1.339	−0.220**	0.064	−0.398	−0.147	1.346
Education level	−0.009	0.059	−0.127	0.104	1.175	0.090	0.064	−0.006	0.246	1.176
Years of experience raising chickens	0.111 *	0.033	0.016	0.145	1.237	0.277 **	0.036	0.130	0.271	1.225
Poultry infection experience	−0.258 **	0.108	−0.806	−0.383	1.310	−0.262 **	0.112	−0.824	−0.382	1.200
Farming operation size	0.520 **	0.053	0.520	0.727	1.153	0.138 **	0.058	0.052	0.279	1.176
Risk perception of poultry infection	0.125 **	0.046	0.031	0.213	1.326	-	-	-	-	-
Risk perception of human infection	-	-	-	-	-	0.110 *	0.055	0.013	0.230	1.257

* *P* < 0.05; ** *P* < 0.01. “-” indicates no data in a given cell. β refers to standardized coefficient. SE refers to standard error. VIF refers to variance inflation factor.

## Appendix A

                **Questionnaire**

**1 Your gender?**

□A. Female   □B. Male

**2 Your age?**

□A. ≤35 years   □B. 36–45 years   □C. 46–55 years   □D. ≥56 years

**3 Your education?**

□A. Primary or below    □B. Junior high school   □C. Senior high school

□D. Three-year college     □E. Undergraduate college or above

**4 Your years of experience raising chickens**

□A. ≤5 years  □B. 6–10 years  □C. 11–15 years  □D. 16–20 years

□E. 21–25 years □F. 26–30 years □G. ≥31 years

**5 Have your chickens ever been infected with A/H5N1 before?**

□A. chickens have been infected with A/H5N1 before

□B. chickens have not been infected with A/H5N1 before

**6 How many chickens do you have on hand now?**

□A. ≤ 300 chickens     □B. 301–1000 chickens

□C. 1001–10,000 chickens     □D. ≥10,001 chickens

**7 How much risk do you feel that your poultry may be infected with A/H5N1?**

□A. No perceived risk whatsoever   □B. Very small risk   □C. Some risk

□D. Relatively high risk    □E. Very high perceived risk

**8 How much risk do you feel that you may be infected with A/H7N9?**

□A. No perceived risk whatsoever   □B. Very small risk   □C. Some risk

□D. Relatively high risk     □E. Very high perceived risk

**9 Please tick √ in the box you think most suitable**

**Familiarity (poultry infection)**	**Familiarity (1–5 gradually increase)**				
1 Familiarity of occurrence reason to poultry infection	□1	□2	□3	□4	□5
2 Familiarity of route of transmission and infectivity to poultry infection	□1	□2	□3	□4	□5
3 Familiarity of cure rate to poultry infection	□1	□2	□3	□4	□5
4 Familiarity of prevention measure to poultry infection	□1	□2	□3	□4	□5
5 Familiarity of death rate to poultry infection	□1	□2	□3	□4	□5
6 Familiarity of overall feelings about poultry infection	□1	□2	□3	□4	□5
**Controllability (poultry infection)**	**Controllability (1–5 gradually increase)**				
1 Controllability of poultry infection	□1	□2	□3	□4	□5
2 Controllability of route of transmission and infectivity to poultry infection	□1	□2	□3	□4	□5
3 Controllability of cure rate to poultry infection	□1	□2	□3	□4	□5
4 Controllability of prevention measure to poultry infection	□1	□2	□3	□4	□5
5 Controllability of death rate to poultry infection	□1	□2	□3	□4	□5
6 Controllability of overall feelings about poultry infection	□1	□2	□3	□4	□5

**10. Please tick √ in the box you think most suitable.**

**Familiarity (human infection)**	**Familiarity (1–5 gradual increase)**				
1 Familiarity of occurrence reason to human infection	□1	□2	□3	□4	□5
2 Familiarity of route of transmission and infectivity to human infection	□1	□2	□3	□4	□5
3 Familiarity of cure rate to human infection	□1	□2	□3	□4	□5
4 Familiarity of prevention measure to human infection	□1	□2	□3	□4	□5
5 Familiarity of death rate to human infection	□1	□2	□3	□4	□5
6 Familiarity of overall feelings about human infection	□1	□2	□3	□4	□5
**Controllability (human infection)**	**Controllability (1–5 gradual increase)**				
1 Controllability of human infection occurrence	□1	□2	□3	□4	□5
2 Controllability of route of transmission and infectivity to human infection	□1	□2	□3	□4	□5
3 Controllability of cure rate to human infection	□1	□2	□3	□4	□5
4 Controllability of prevention measure to human infection	□1	□2	□3	□4	□5
5 Controllability of death rate to human infection	□1	□2	□3	□4	□5
6 Controllability of overall feelings about human infection	□1	□2	□3	□4	□5

**11. What preventive measures have you taken in your routine husbandry practices to prevent A/H5N1 infection in poultry? Put “√” in the appropriate box.**

**Preventive** **Measures**	**Never Adopted**	**Sometimes Adopted**	**Often Adopted**	**Usually Adopted**	**Adopted All the Time**
1 Closed doors and windows all the time in winter	□	□	□	□	□
2 Prevented mice and flies in the poultry house	□	□	□	□	□
3 Conducted all-in and all-out method	□	□	□	□	□
4 Prevented contact with neighbors’ poultry	□	□	□	□	□
5 Prevented contact with wild poultry	□	□	□	□	□
6 Frequently cleaned floors and chicken cages	□	□	□	□	□
7 Disinfected staff, vehicles, and goods entering the poultry house	□	□	□	□	□
8 Quarantined newly-purchased poultry	□	□	□	□	□
9 Continuously disinfected with chicken in cage 2–3 times weekly	□	□	□	□	□

**12. What preventive measures have you taken in your routine husbandry practices to prevent A/H7N9 infection in yourself? Put “√” in the appropriate box.**

**Preventive** **Measures**	**Never Adopted**	**Sometimes Adopted**	**Often Adopted**	**Usually Adopted**	**Adopted All the Time**
1 Wear gloves	□	□	□	□	□
2 Wear protective clothes	□	□	□	□	□
3 Wear face mask	□	□	□	□	□
4 Wear protective hat	□	□	□	□	□
5 Wear protective shoes	□	□	□	□	□
6 Washing hands after touching dead poultry	□	□	□	□	□
7 Washing hands after touching poultry feces	□	□	□	□	□
